# Exonic *CLDN16* mutations associated with familial hypomagnesemia with hypercalciuria and nephrocalcinosis can induce deleterious mRNA alterations

**DOI:** 10.1186/s12881-018-0713-7

**Published:** 2019-01-08

**Authors:** Ana Perdomo-Ramirez, Marian de Armas-Ortiz, Elena Ramos-Trujillo, Lorena Suarez-Artiles, Felix Claverie-Martin

**Affiliations:** Unidad de Investigación, Hospital Nuestra Señora de Candelaria, Carretera del Rosario 145, 38010 Santa Cruz de Tenerife, Spain

**Keywords:** Missense mutation, Pre-mRNA splicing, Exonic splicing enhancer, Minigene, Splicing defects, Bioinformatics analysis, Claudin-16

## Abstract

**Background:**

Familial hypomagnesaemia with hypercalciuria and nephrocalcinosis type 1 is an autosomal recessive disease characterized by excessive renal magnesium and calcium excretion, bilateral nephrocalcinosis, and progressive chronic renal failure. This rare disease is caused by mutations in *CLDN16* that encodes claudin-16, a tight-junction protein involved in paracellular reabsorption of magnesium and calcium in the renal tubule. Most of these variants are located in exons and have been classified as missense mutations. The functional consequences of some of these claudin-16 mutant proteins have been analysed after heterologous expression showing indeed a significant loss of function compared to the wild-type claudin-16. We hypothesize that a number of *CLDN16* exonic mutations can be responsible for the disease phenotype by disrupting the pre-mRNA splicing process.

**Methods:**

We selected 12 previously described presumed *CLDN16* missense mutations and analysed their potential effect on pre-mRNA splicing using a minigene assay.

**Results:**

Our results indicate that five of these mutations induce significant splicing alterations. Mutations c.453G > T and c.446G > T seem to inactivate exonic splicing enhancers and promote the use of an internal cryptic acceptor splice site resulting in inclusion of a truncated exon 3 in the mature mRNA. Mutation c.571G > A affects an exonic splicing enhancer resulting in partial skipping of exon 3. Mutations c.593G > C and c.593G > A disturb the acceptor splice site of intron 3 and cause complete exon 4 skipping.

**Conclusions:**

To our knowledge, this is the first report of *CLDN16* exonic mutations producing alterations in splicing. We suggest that in the absence of patients RNA samples, splicing functional assays with minigenes could be valuable for evaluating the effect of exonic *CLDN16* mutations on pre-mRNA splicing.

**Electronic supplementary material:**

The online version of this article (10.1186/s12881-018-0713-7) contains supplementary material, which is available to authorized users.

## Background

**Fig. 1:**
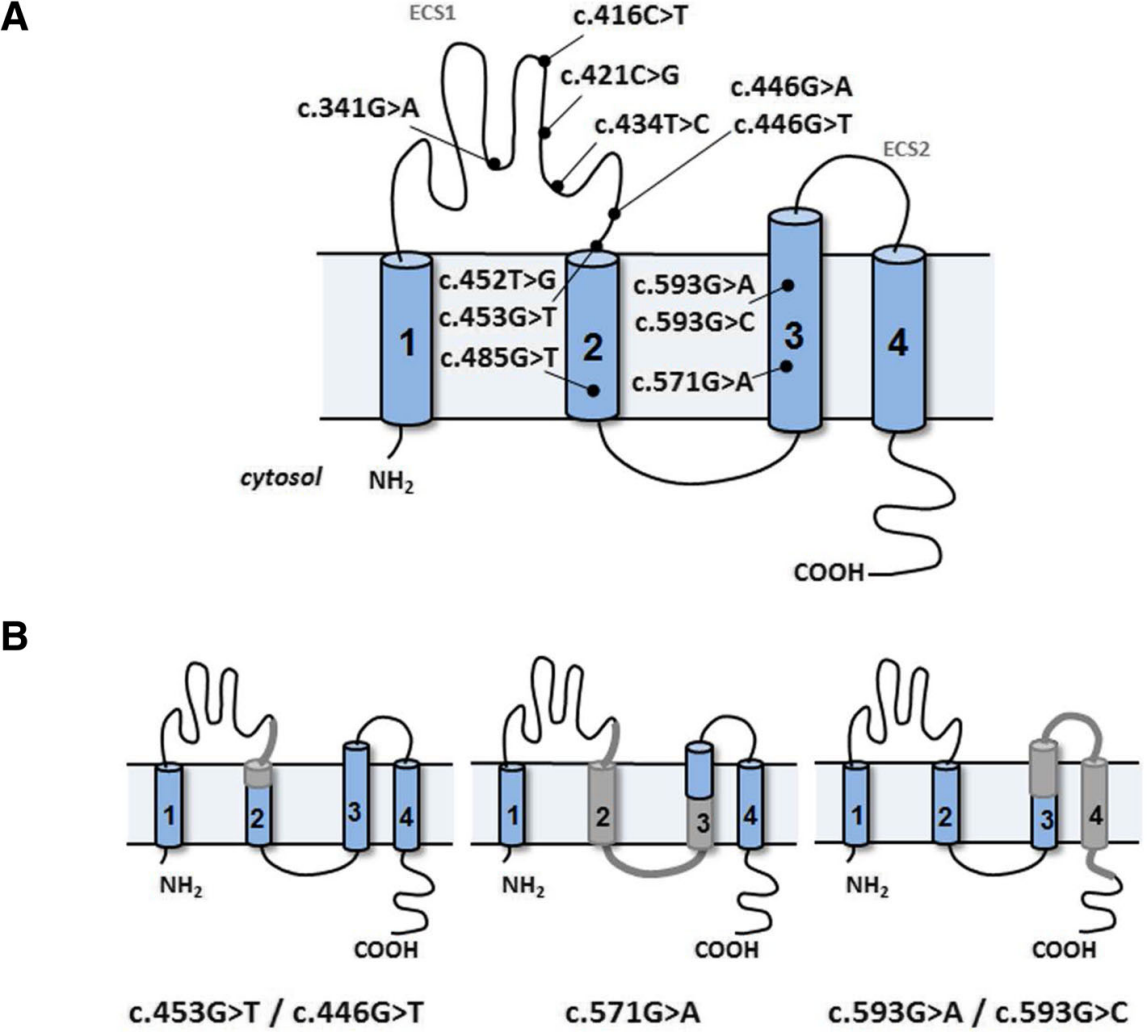
Diagrammatic representations of claudin-16. **a** The protein contains four TMDs (1–4) and two ECSs (1–2). ECS1 contains the ion selectivity filter while ECS2 is involved in claudin–claudin interactions. The location of the mutations included in this study is indicated. **b** Hypothetical protein structures of mutant claudin-16 proteins translated from the aberrant transcripts detected in Fig. 3a. Truncated exon 3 induced by mutations c.446G > T and c.453G > T would result in loss of the terminal part of ECS1 and part of TMD2. Skipping of exon 3 produced by mutation c.571G > A would cause loss of the terminal part of ECS1, TMD2, the cytoplasmic region and part of TMD3. Skipping of exon 4 induced mutations c.593G > A and c593G > C would result in loss of part of TMD3, ECS2, TMD4, and part of the cytoplasmic C terminus

Pathogenic variants in *CLDN16* and *CLDN19* genes cause a rare autosomal recessive disease named Familial Hypomagnesaemia with Hypercalciuria and Nephrocalcinosis (FHHNC; MIM #248250 and #248190) [1–3]. Patients present renal Mg^++^ and Ca^++^ wasting, and develop nephrocalcinosis, chronic kidney failure and amelogenesis imperfecta [4–6]. Patients with mutations in *CLDN19* also display severe ocular defects including myopia magna, nystagmus and macular colobamata [2, 7, 8]. *CLDN16* and *CLDN19* encode the tight junction (TJ) proteins claudin-16 and claudin-19, respectively, which regulate the paracellular reabsorption of Mg^++^ and Ca^++^ in the kidney [9–11]. These claudins are members of a family of membrane proteins that contain four transmembrane domains (TMDs) and two extracellular segments (ECSs) [12] (Fig. 1a).

According to the Human Gene Mutation Database Professional 2018.1, 62 *CLDN16* mutations have been identified in FHHNC patients, 42 of which are missense mutations [13]. These mutations are mainly located in the two ECSs but a few affect the TMDs and the cytoplasmic regions. The functional consequences of some of these *CLDN16* mutants have been analysed after heterologous expression in cultured cells [9, 14, 15]. Most mutant proteins display normal trafficking to the cell membrane, others are retained inside the cell (endoplasmic reticulum, Golgi apparatus or lysosomes), and a few are not expressed. The mutant proteins that are properly targeted to the TJ usually show a significant loss of function compared to the wild-type (WT) claudin-16 [9, 15]. Also, using coexpression studies, Hou and colleagues have shown that claudin-16 interacts with claudin-19 and that this interaction confers cation selectivity to the TJ in a synergistic manner [11]. Introduction of pathogenic changes in either *CLDN16* or *CLDN19* disrupts this interaction and abolished the synergistic effect [11].

However, none of these presume missense mutations have been tested for their effect in precursor-mRNAs (pre-mRNA) splicing. Over the last 20 years it has become evident that a significant fraction of exonic variants, including missense and synonymous mutations, can cause disease by disrupting this process [16–18]. In addition to protein coding information, exons include sequences required for correct pre-mRNA splicing. Exonic mutations can modify either splice sites or splicing regulatory elements, such as exonic splicing enhancers (ESEs) or exonic splicing silencers (ESSs), resulting in defective mRNAs. ESEs and ESSs are generally located within exon ends and function by interacting with trans-factors, mainly serine/arginine-rich (SR) proteins and heterogeneous nuclear ribonucleoproteins (hnRNPs) [19–22].

The ideal approach to identify splicing alterations is the analysis of RNA obtained from the patient. However, this type of sample is not always accessible. Minigene analysis has become an alternative methodology to initially test whether a particular mutation affects pre-mRNA splicing [23–26]. For this purpose, a fragment of the specific gene containing exons and intron sequences is cloned in an expression vector and the mutations of interest are introduced individually by site directed mutagenesis. These constructs are transfected into cells, the RNA is analysed by reverse transcription polymerase chain reaction (RT-PCR), and the splicing patterns are compared with those of the controls.

In previous studies, we have tested the impact on splicing of presumed missense and synonymous mutations associated with other renal hereditary diseases by minigene assays and found abnormal splicing patterns for some mutations [27–29]. Here, we selected and assayed in the minigene system twelve previously described *CLDN16* missense mutations that are located relatively close to the exon ends. These include the predominant mutation in patients from Germany and Eastern Europe, c.453G > T, p.(L151F), and the recurrent disease-causing variant in North African families, c.416C > T, p.(A139V) [7, 30], both of which are due to a founder effect. Our results showed that five mutations induce major splicing defects resulting in skipping of an exon or incorporation of an incomplete exon in the mature mRNA.

## Methods

We selected twelve *CLDN16* pathogenic mutations previously identified in exons 2, 3 and 4 (Table 1). These mutations are located within 70 nucleotides from the exon ends, since these regions are known to be rich in splice regulatory elements, and because splice-disrupting mutations have a tendency to occur mainly there [16, 31–33]. All these mutations had been predicted to cause a change of amino acid in the claudin-16 protein. The effect of these gene variants on pre-mRNA splicing was examined using a minigene assay, as RNA from patients was unavailable.

**Table 1:** Claudin-16 exonic mutations selected for this study and their effects

Mutation	Reference	Exon	Position in exon^a^	Amino acid change prediction		Protein function^b^	Minigene result
				SIFT (score)	PolyPhen (score)		
c.341G > A; p.(R114Q)	[15]	2	+ 17	Tolerated (0.25)	Possibly damaging (0.748)	Partial loss	No effect
c.416C > T; p.(A139V)	[41]	2	-12	Damaging (0.01)	Possibly damaging (0.774)	–	No effect
c.421C > G; p.(H141D)	[42]	2	-7	Tolerated (1.00)	Probably damaging (0.999)	Partial loss	No effect
c.434 T > C; p.(L145P)	[42]	3	+ 7	Damaging (0.00)	Probably damaging (0.972)	Complete loss	No effect
c.446G > A; p.(R149Q)	[43]	3	+ 19	Damaging (0.03)	Probably damaging (0.999)	–	No effect
c.446G > T; p.(R149L)	[30]	3	+ 19	Damaging (0.00)	Probably damaging (0.999)	Complete loss	Truncated exon 3
c.452 T > G; p.(L151W)	[42]	3	+ 25	Damaging (0.00)	Probably damaging (0.998)	Partial loss	No effect
c.453G > T; p.(L151F)	[42]	3	+ 26	Damaging (0.00)	Probably damaging (0.985)	Partial loss	Truncated exon 3
c.485G > T; p.(G162V)	[41]	3	+ 58	Damaging (0.01)	Probably damaging (0.989)	–	No effect
c.571G > A; p.(G191R)	[1]	3	−22	Damaging (0.00)	Probably damaging (1.000)	Partial loss	Exon 3 skipping
c.593G > A; p.(G198D)	[1]	4	+ 1	Damaging (0.00)	Probably damaging (0.994)	Complete loss	Exon 4 skipping
c.593G > C; p.(G198A)	[30]	4	+ 1	Tolerated (0.07)	Probably Damaging (0.961)	–	Exon 4 skipping

^a^Position in relation to the 3′ (+) or 5′ (−) splice site

^b^Data on mutant claudin-16 function are from references [9, 15]

### Minigene construction and site-directed mutagenesis

*CLDN16* minigene constructs were derived from expression vector pET01 (MoBITec, Göttingen, Germany), and were basically generated as we previously described [29]. Briefly, exons 2, 3 and 4 of *CLDN16* and the flanking intronic sequences were amplified by PCR from a control genomic DNA using specific primers that contain restriction sites for *Xho*I, *Bam*HI or *Xba*I at their 5′ ends (Additional file 1: Table S1). These primers were design using the web-based sources Primer3 [34] and GeneRunner (Hastings Software, Inc., San Francisco, CA, USA). The PCR products were then digested with the proper restriction enzymes and cloned into the *Xho*I/*Xba*I- or *Bam*HI/*Xba*I-digested pET01 vector.

Most sequence changes were introduced in the WT minigenes by site-directed mutagenesis using the Quik-Change II Site-Directed Mutagenesis Kit (Agilent Technologies, Santa Clara, CA, USA) according to the manufacturer’s instructions. Primers were designed to create the mutations of interest using QuikChange Primer Design application (https://www.genomics.agilent.com/primerDesignProgram.jsp) (Additional file 1: Table S1). Minigene constructs with mutations c.485G > T, p.(Gly162Val), and c.571G > A, p.(Gly191Arg), were assembled by PCR amplification from patients’ genomic DNA and cloning into the pET01 vector. These two genomic samples had been obtained in the past from FHHNC patients referred to our hospital for genetic analysis (unpublished results). We confirmed the fidelity of all minigenes by DNA sequencing on a 3500 Series Genetic Analyzer (Applied Biosystems, Foster City, CA, USA). Certain samples were sent out to Macrogen Europe (Amsterdam, The Netherlands) for their analysis.

### Minigene splicing assays

In order to assess the effect on pre-mRNA splicing of *CLDN16* exonic mutations, we carried out a functional assay based on the comparative study of the splicing patterns of WT and mutant minigenes. These minigenes were transfected into COS7 cells using JetPRIME® (Polyplus Tranfection, Illkirch, France), according to the manufacturer’s instructions. Cells were harvested after incubation for 48 h and total RNA was isolated using the High Pure RNA Isolation Kit (Roche, Basel, Switzerland). RT-PCR was carried out using iScript™ Select cDNA Synthesis Kit (Bio-Rad, Hercules, CA, USA) with random primers, in accordance with manufacturer’s instructions. The resulting cDNA was amplified by PCR using forward (PCR Primer 2) and reverse (PCR Primer 3) primers complementary to the 5′ and 3′ exons of the pET01 vector (MoBITec). Products were separated by agarose gel (1.5%) electrophoresis together with the PCR 100 bp Low Ladder (Sigma-Aldrich) as molecular weight marker, and visualized in a Gel Doc EZ Imager (Bio-Rad) after staining with GelRed (Biotoium, Inc., Fremont, CA, USA). DNA bands were recovered from the agarose gels using the GenElute™ Gel Extraction Kit (Sigma-Aldrich) and analysed by DNA sequencing.

### In silico analysis

As a complementary investigation, we tested in silico the impact of mutations on splicing regulatory elements and splice sites using the following bioinformatics tools. Human Splicing Finder v3.1 (HSF) incorporates matrices reported by different groups to identify exonic and intronic motifs [35]. SPANR analyses nucleotides motifs to predict the splicing effects of genetic variants on an exon and its flanking intronic regions [36]. MutPred Splice v1.3.2 integrates multiple characteristics of mutations to instruct a machine learning-based model to predict the effect of exonic variants on pre-mRNA splicing [37]. NNSplice v0.9 is an integrated tool to predict splice sites and can also be used to assess the effect of gene variants on splicing [38]. To predict the effects of amino acid substitutions on proteins we used the following tools: PolyPhen-2, that predicts the potential impact of an amino acid substitution on the structure and function of a human protein using physical and comparative considerations [39], and SIFT that predicts whether an amino acid substitution affects protein function based on sequence homology and the physical properties of amino acids [40]. Default settings were employed for all programs.

## Results

**Fig. 2:**
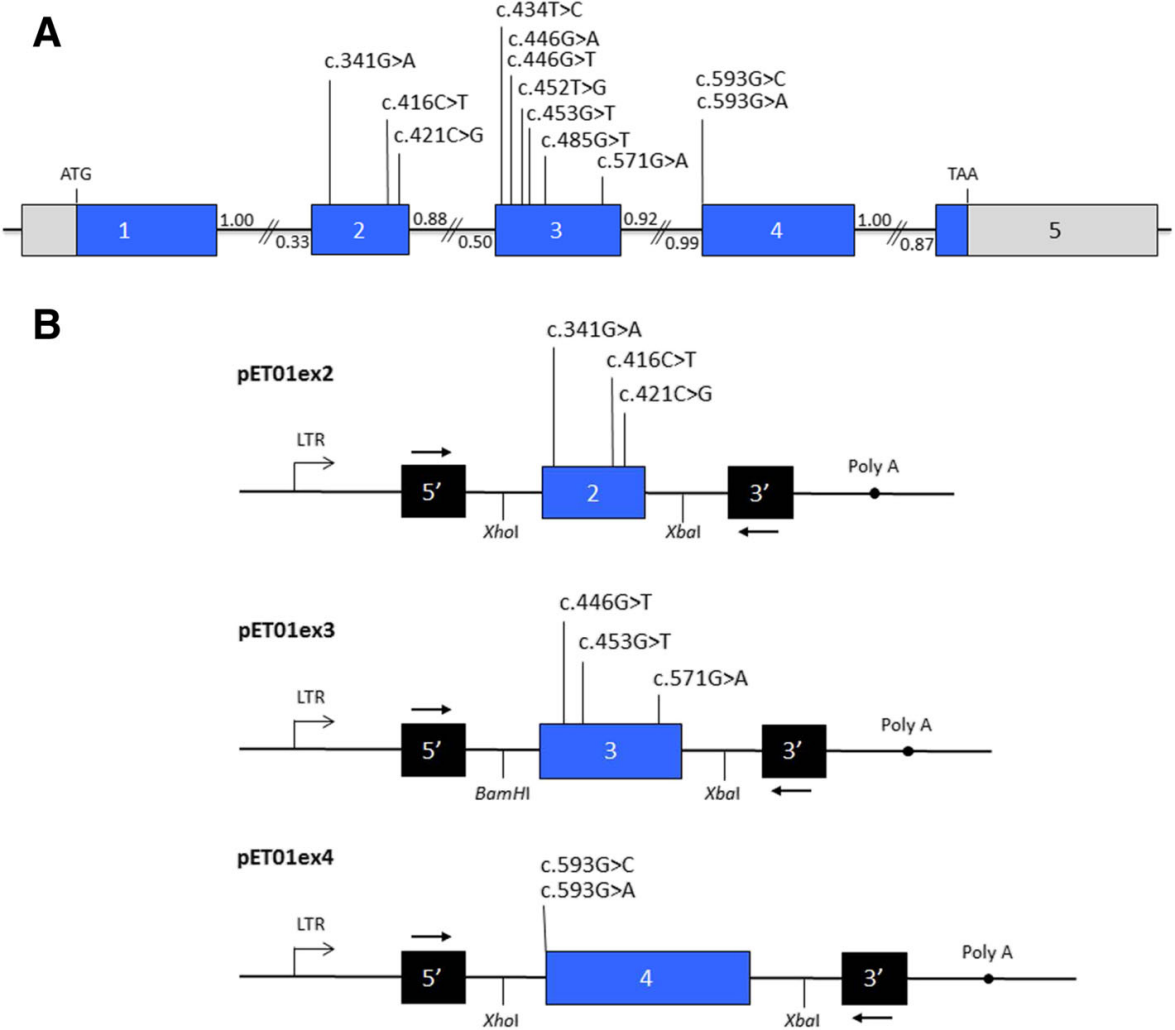
Location of presume missense mutations analysed in this study and schematic representation of *CLDN16* minigenes. **a** Boxes represent the five coding exons and black lines in between indicate intron sequences. Coding and non-coding regions appears in blue and grey, respectively. Exons and introns sizes are not at scale. Small numbers indicate NNSplice scores of donor and acceptor splice sites. **b** Structure of *CLDN16* minigenes used in the splicing reporter assay. Blue and black boxes represent *CLDN16* exons and 5′ and 3′ exons of the shuttle vector pET01, respectively. Black lines indicate intron sequences. The minigenes were constructed by introducing a *CLDN16* genomic fragment containing exons 2, 3 or 4 and flanking intronic sequences into the vector as described in Materials and Methods. The position of each mutation and the restriction sites used for cloning are indicated. Arrows above and below the vector exons represent primers used in the RT-PCR assays. LTR, long terminal repeat promoter of the Rous sarcoma virus; Poly A, polyadenylation site. The minigenes will express a poly-A+ RNA containing the spliced exons

**Fig. 3:**
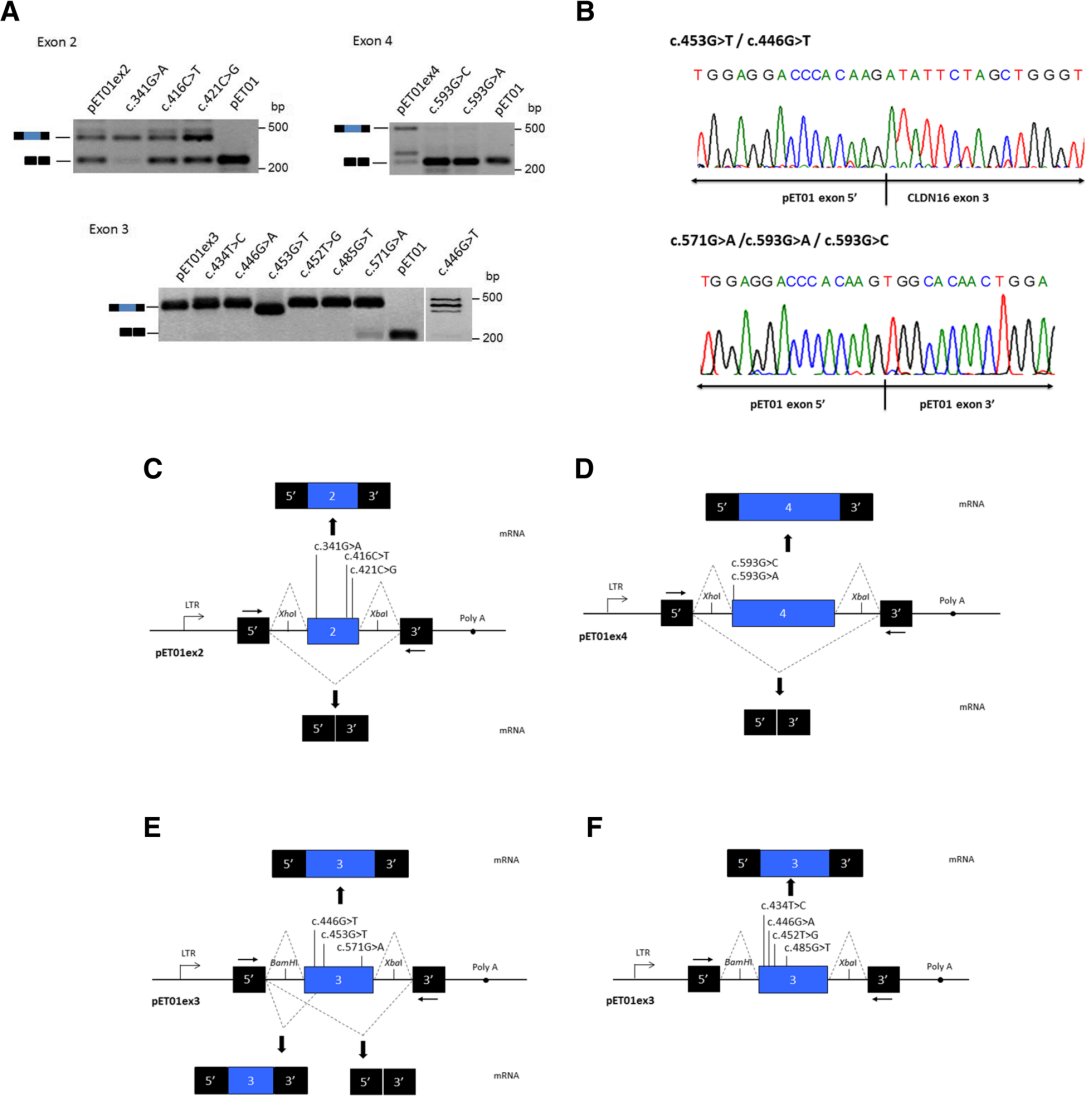
Minigene assay of *CLDN16* exonic mutations revealed altered pre-mRNA splicing. **a** RT-PCR fragments produced by minigenes and separated by agarose gel electrophoresis. Wild-type and mutant pET01ex2, pET01ex3 and pET01ex4 constructs were transfected into COS7 cells, and the mRNAs were analysed as described in Material and Methods. All assays were performed in triplicate. The identities of the RT-PCR products are illustrated schematically on the left of each panel. **b** Electropherograms of anomalous RT-PCR fragments produced by minigenes containing the mutations indicated. **c-f** Schematic representation of minigene splicing in the presence and absence of mutations. The location of mutations is indicated. Mutations c.453G > T and c.446G > T activate a cryptic acceptor splice site internal to exon 3 that induces inclusion of a truncated exon 3. Mutation c.571G > A produced a major band corresponding to the normally spliced transcript together with a minor band that correspond to skipping of exon 3. Both c.593G > C and c.593G > A inactivate the splice site and result in exon 4 skipping

In this study, we used a minigene approach to analyse twelve point mutations detected in exons 2, 3 and 4 of the *CLDN16* gene for their effect on pre-mRNA splicing (Table 1, Fig. 2a). These mutations, all localized within 70 nucleotides from the exon ends, were previously identified in different families with FHHNC and were reported as missense mutations [1, 14, 30, 41–43]. Minigenes contained *CLDN16* genomic inserts of 250 bp, 369 bp and 368 bp, respectively, with the following organization (Fig. 2b): pET01ex2, intron 1 (97 bp)-exon 2 (103 bp)-intron 2 (50 bp); pET01ex3, intron 2 (117 bp)-exon 3 (169 bp)-intron 3 (83 bp); pET01ex4, intron 3 (132 bp)-exon 4 (192 bp)-intron 4 (44 bp). Each minigene construct was transfected into COS7 cells, and the RNA was purified and analysed as indicated in the methods section. We found that the three WT constructs produced transcripts containing the corresponding exon, but for two of them, pET01ex2 and pET01ex4, we also observed expression of transcripts missing the respective exon (Fig. 3a). Furthermore, minigene pET01ex4 produced a third band that runs above the band missing exon 4, which we were unable to characterize. Five of the mutations analysed generated altered splicing products. In three of them, c.453G > T, c.593G > C and c.593G > A, the altered splicing was total, and in the other two, c.571G > A and c446G > T, the effect was partial implying that these mutations still produced normal transcripts.

Three mutations located in exon 2 were analysed (Table 1, Fig. 2b). Mutation c.341G > A affects nucleotide + 17 from the 5′ end of exon 2, while mutations c.421C > G and c.416C > T affect positions − 7 and − 12, respectively, upstream from the 3′ end of exon 2. None of these mutations altered the splicing pattern of exon 2 compared to the WT (Fig. 3a); control and mutant minigenes produced both the expected splicing product containing exon 2, and a band that corresponds to the skipping of this exon (Fig. 3c), which could be due the low score (0.33, according to NNSplice) of the acceptor splice site of intron 1. However, it should be noted that in the splice pattern of mutations c.341G > A and c.421C > G the ratio between exon inclusion and exon skipping showed a major shift towards exon inclusion compared to the WT and mutation c.416C > T (Fig. 3a). Mutation c.453G > T, which affects nucleotide position + 26 of exon 3, produced a single band that was shorter than the band appearing in the control minigene (Fig. 3a). Sequencing analysis showed that the shorter band corresponds to exon 3 with a 39-nucleotide deletion at the 5′ end. Interestingly, we detected the activation of an internal cryptic 5′ splice site (acceptor) (AG) at c.466 resulting in the shorter transcript (Fig. 3e). A similar results was obtained for mutation c.446G > T, however, in this case two more bands were observed, one corresponding to the inclusion of an intact exon 3 and a larger one that we were unable to characterized (Fig. 3a and e). Therefore, mutations c.453G > T and c.446G > T induce inclusion of a truncated exon 3 in the mRNA. We also analysed these two mutations with the bioinformatics tool HSF, and found that both mutations inactivate a potential ESE site within the sequence GTAACTCGAGCGTTGATGAT located close to the 5′ end of exon 3 (the nucleotides affected by the respective mutations are underlined). The minigene assay for mutation c.571G > A, located on the other end of exon 3 at position − 22, showed a partial effect on splicing, with the presence of a major band corresponding to the normally spliced transcript together with a minor band that corresponds to the skipping of exon 3 (Fig. 3a and e). Analysis of both products by DNA sequencing confirmed these predictions and the presence of the c.571G > A mutation in the larger band. According to HSF, mutation c.571G > A was predicted to affect an ESE site in the sequence TGTTGCTGGAGCC in exon 3. Since none of these three mutations reduced the strength of the canonical splice sites and taking into account the results of the functional splicing assay, we suggest that their effect on splicing is due to the alteration of ESEs. The other four mutations located in exon 3, c.434 T > C, c.446G > A, c.425 T > G and c.485G > T showed no effect on the splicing pattern compared to the WT minigene (Fig. 3a and f).

Two mutations, c.593G > A and c.593G > C, located in exon 4 were selected for the minigene analysis. Both mutations affect the first nucleotide of the exon, which lies downstream of the conserved AG dinucleotide of the acceptor splice site of intron 3 (Fig. 2b). Both mutations produced a main band that run together with the product of the pET01 vector (Fig. 3a and d). DNA sequencing of this band showed the joining of the two exons from the vector (Fig. 3b). The band corresponding to normal exon 4 inclusion was not produced by the mutant minigenes. Therefore, these two mutations inactivate the acceptor splice site and result in skipping of exon 4.

Taking into account the results obtained with the minigene analysis, we then examined these twelve mutations with three different prediction algorithms, Human Splicing Finder, SPANR and MutPred Splice, in order to investigate concordance or discrepancy between the two approaches. The results of this analysis are shown in Additional file 2: Table S2. None of the splicing alterations detected in our minigene assay was predicted with all the bioinformatics tools. Splicing alterations for mutations c.446G > T and c.485G > T were predicted correctly with SPANR and HSF and with MutPred Splice and HSF, respectively, while the other three mutations were predicted to affect splicing by only one of the bioinformatics tools. Predictions of splicing alterations with MutPred Splice and SPANR were correct for 3 and 2 out of 5 mutations, respectively, whereas HSF gave the highest number of false positives (5). According to MutPred Splice, mutations c.593G > C and c.593G > A, both affecting the first nucleotide of exon 4, which is considered part of the acceptor splice site, were predicted to affect splicing in agreement with the minigene results. But remarkably, analysis of these mutations with HSF and SPANR suggested no significant splicing effect. Also, analysis with the frequently used algorithm NNSplice predicted that the canonical acceptor splice site of intron 3 (score 0.99) was not significantly altered by mutations c.593G > C (0.97) and c.593G > A (0.98).

## Discussion

Pre-mRNA splicing is a key process in eukaryotic gene expression by means of which introns are removed and exons are pasted successively. The splicing process is facilitated by a ribonucleotide complex, called spliceosome, which interacts with specific RNA sequences at the exon-intron boundaries to control precisely and efficiently intron excision and exon ligation and generate correct mature mRNAs [44]. The incorrect recognition of exon–intron boundaries or the failure to eliminate an intron produces abnormal mRNAs that either encode faulty protein or are degraded. Exon recognition needs specific signals at the 5′ and 3′ splice sites, the polypyrimidine tract, the branch point, and splicing regulatory elements such as exonic and intronic splicing enhancers and silencers to which RNA binding proteins attach [16, 45]. Mutations in these cis-motifs can disrupt the splicing process and induce disease phenotypes in humans. In fact, it has been estimated that approximately 25% of exonic disease-causing mutations affect pre-mRNA splicing [46, 47].

The purpose of the present study was to evaluate the effect of a set of exonic *CLDN16* mutations that cause FHHNC, a rare disease associated with progressive renal failure, on pre-mRNA splicing using a minigene-based approach. Our assumption was that some *CLDN16* mutations initially reported as missense could also affect splicing. Before this study, no such analyses had been reported for *CLDN16* mutations. For this purpose we generated hybrid minigenes containing either WT or mutant *CLDN16* sequences. Assessment of the splicing pattern of both WT and mutant minigenes allowed us to establish the effect of each mutation on the splicing. Notably, among the twelve exonic mutations examined, we identified 5 mutations that altered splicing; three mutations caused exon skipping and the other two resulted in incorporation of a truncated exon.

Mutations c.453G > T and c.446G > T, located in exon 3 of *CLDN16*, have been reported as missense mutations p.(L151F) and p.(R149L), respectively [30, 42]. Both mutations affect highly conserved amino acid residues in the ECS1 of claudin-16 (Fig. 1a). Mutation c.453G > T is the most frequent *CLDN16* disease-causing variant detected in FHHNC patients from Germany and Eastern Europe [30]. Our minigene analysis showed that these mutations induce the generation of a transcript containing a truncated exon 3. The level of the splicing alteration induced by mutation c.446G > T was much lower than the one observed for c.453G > T. Both mutations were found to affect ESE elements and are close to the acceptor splice site of intron 2, which has a weak score (0.50 according to NNSplice). As has been shown in other cases, it is possible that this splice site needs the support of the neighboring ESEs in order to be used [16, 48]. Mutations c.453G > T and c.446G > T eliminate this support. The effect on the claudin-16 protein of joining exon 2 and the truncated exon 3 would be the loss of 14 amino acids (L-K-L-V-V-T-R-A-L-M-I-T-A-D) and the inclusion of a new tyrosine in the mutant protein (Additional file 3: Figure S1). The region affected includes part of ECS1 and part of TMD2 of claudin-16 (Fig. 1b).

Mutation c.571G > A, also located in exon 3, has been reported as missense mutation p.(G191R) that affects TMD3 of claudin-16 [1]. The results obtained with the minigene analysis show that this mutation has also a partial effect on splicing consisting in skipping of the exon. The subsequent joining of exons 2 and 4 would cause the loss of 56 amino acids and the inclusion of a new cysteine in the translated mutant claudin-16 protein (Additional file 3: Figure S1). Consequently, this mutant protein would lack the entire TMD2 and part of TMD3 (Fig. 1b).

Mutations c.593G > C and c.593G > A, located in exon 4, have been reported as missense mutations p.(G198D) and p.(G198A), respectively [1, 42]. These amino acid changes affect TMD3 of claudin-16 (Fig. 1a). However, our results of the minigene analysis showed that both mutations produce transcripts lacking exon 4. These mutations affect the first nucleotide of exon 4, which is actually an integral part of the acceptor splice site; therefore we believe that they inactivate it. Sequence analysis suggests that the fusion of exons 3 and 5 does not alter the open reading frame (Additional file 3: Figure S1). The subsequent joining of exons 3 and 5 would cause the loss of 65 amino acids and the inclusion of a new aspartic acid residue in the translated mutant claudin-16 protein (Additional file 3: Figure S1). Consequently, these mutant proteins would lack part of TMD3, the entire TMD4 and part of the cytoplasmic carboxy terminus (Fig. 1b).

*CLDN16* produces only one recognized transcript containing five exons. Therefore, we could not find a reasonable explanation for the presence of the uncharacterized band produced by the minigene containing WT exon 4. Interestingly, this band was not generated by the two minigenes containing exon 4 mutations. On the other hand, minigenes containing the WT exon 2 or its mutants produced, in addition to the expected band containing exon 2, a band lacking the exon. This could be due to the weak acceptor splice site present in intron 1. Perhaps, the cell line we used to transfect the minigenes lacks essential splicing factors present in the human kidney cells that induce the incorporation of exon 2 in the mRNA.

Even though the splicing defects caused by these five mutations do not imply any alteration in the reading frame of *CLDN16* they would cause drastic losses in the claudin-16 protein. Consequently, we consider mutations c.453G > T, c.593G > C and c.593G > A damaging because of the total effect they produce in splicing. The clinical significance of mutations c.571G > A and c.446G > T with regard to the splicing effect would be more difficult to predict since they induce partial splicing defects. The splicing effect caused by mutation c.571G > A is small and the majority of transcripts generated by this minigene contain the mutation, which produces a partial loss of function in the claudin-16 protein [9] (Table 1). On the other hand, mutation c.446G > T, which abolishes completely claudin-16 function [9] (Table 1), produces more impact on splicing than c.571G > A. Therefore, the pathogenic effect of these two mutations may be due to the combination of the presence of the normal-length transcript bearing the missense mutation and the altered splicing. Mutations c.341G > A and c.421C > G seem to increase exon 2 inclusion in the minigene assay (Fig. 3a). It is difficult to predict the effect of this splicing modification, but a few cases of mutation-induced exon inclusion have been reported in other genes and some can have important clinical consequences [49–51].

Previous results by different groups indicate that the informatics tools available to predict the effect of variants on splicing are not very reliable [27–29, 52, 53]. In the present study, we used the results obtained with the minigene analyses to assess the predictive capability of three bioinformatics tools at differentiating splicing mutations. Our findings showed that the analysis with MutPred Splice, SPANR and Human Splicing Finder were contradictory and agreed only partially with the results of the minigene analysis. Furthermore, we found low correlation between the predictions obtained with these tools. Surprisingly, bioinformatics analysis with NNSplice, SPANR and HSF predicted that neither mutation c.593G > A nor c.593G > C, which affect the first nucleotide of exon 4, would abolish the function of the acceptor splice site. Although our findings with minigene assays will need to be confirmed analysing RNA from patients, results of several studies have indicated nearly 100% concordance between the results obtained with the analysis of patients’ RNA and those from cells transfected with minigenes [24–26].

## Conclusions

Our results revealed that five presumed missense mutations, associated with FHHNC and located in exons 3 and 4 of *CLDN16*, altered pre-mRNA splicing. We suggest that mutations c.453G > T and c.446G > T disrupt potential ESEs, which may be needed for splicing at the canonical acceptor site, and activate an internal acceptor splice site, thus resulting in inclusion of a truncated exon 3 in the mRNA. Mutation c.571G > A affects an ESE site resulting in partial skipping of exon 3. Mutations c.593G > C and c.593G > A disturb the acceptor splice site of intron 3 and result in exon 4 skipping. Thus, some presume missense mutations are erroneously assumed to cause defects on the protein as a consequence only of the amino acid substitution. Since a growing number of patients will be subject to DNA sequence analysis for diagnostic reasons, we need to take into account that these exonic mutations may also cause aberrant splicing. To our knowledge, this is the first description of exonic mutations producing an impact on *CLDN16* splicing. Furthermore, these examples support the notion that in the absence of patient RNA samples, minigene analysis could be a valuable tool for assessing the effect of exonic *CLDN16* mutations on pre-mRNA splicing.

## Additional files

Additional file 1: **Table S1.** Primers used in the construction of the minigene and the site directed mutagenesis. (PDF 102 kb)

Additional file 2: **Table S2.** Bioinformatics predictions of splicing defects for *CLDN16* exonic mutations and comparison with experimental results. (PDF 94 kb)

Additional file 3: **Figure S1.** Precise nature of aberrant transcripts as deduced by DNA sequences and predicted exon fusions. (PDF 1807 kb)

## Abbreviations

ECS: extracellular segment
ESE: exonic splicing enhancer
ESS: exonic splicing silencer
FHHNC: familial hypomagnesaemia with hypercalciuria and nephrocalcinosis
hnRNPs: heterogeneous nuclear ribonucleoproteins
HSF: Human Splicing Finder
MIM: Mendelian Inheritance in Man
RT-PCR: reverse transcription polymerase chain reaction
SR: serine/arginine
TJ: Tight junction
TMD: transmembrane domain
WT: wild-type
