# Supplementary material for: Exonic CLDN16 mutations associated with familial hypomagnesemia with hypercalciuria and nephrocalcinosis can induce deleterious mRNA alterations
Source: BMC Med Genet. 2019 Jan 8;20:6. doi: 10.1186/s12881-018-0713-7 (PMC6325764; doi:10.1186/s12881-018-0713-7)
Supplement: Supplementary file 1 — Table S1. Primers used in the construction of the minigene and the site directed mutagenesis. (PDF 102 kb) [file 12881_2018_713_MOESM1_ESM.pdf]

Table S1. Primers used in the construction of the minigene and the site directed mutagenesis

	Primer (5′-3′)	
	PCR amplification	*Size*
C16 E2 F	TTATCTCGAGTGCTATCAAAC	270 pb
C16 E2 R	TATTTCTAGATTCTGTCCCTTTT	
C16 E3 F	TTATGGATCCTTTACCGGAGG	389 pb
C16 E3 R	TATTTCTAGATGAGCAGCTTC	
C16 E4 F	TTATCTCGAGTTGCCCATGAA	388 pb
C16 E4 R	TATTTCTAGACGATAGTGGAG	
	Site Directed Mutagenesis	
p.(R114Q) F	GAGCACAAAATGCCAAGGCCT	
p.(R114Q) R	CCCACCAGAGGCCTTGGCATT	
p.(A139V) F	GTACGATTCCATACTTGTGGAGCATCCCTGTACGT	
p.(A139V) R	ACGTACAGGGATGCTCCACAAGTATGGAATCGTAC	
p.(H141D) F	TTCCATACTTGCGGAGGATCCCTGTACGTATGC	
p.(H141D) R	GCATACGTACAGGGATCCTCCGCAAGTATGGAA	
p.(L145P) F	GTCTTCCAGTGAAGCCGGTGGTAACTCGAGC	
p.(L145P) R	GCTCGAGTTACCACCGGCTTCACTGGAAGAC	
p.(R149Q) F	GAAGCTGGTGGTAACTCAAGCGTTGATGATTACTG	
p.(R149Q) R	CAGTAATCATCAACGCTTGAGTTACCACCAGCTTC	
p.(R149L) F	GAAGCTGGTGGTAACTCTAGCGTTGATGATTACTG	
p.(R149L) R	CAGTAATCATCAACGCTAGAGTTACCACCAGCTTC	
p.(L151W) F	GGTGGTAACTCGAGCGTGGATGATTACTGCAGATA	
p.(L151W) R	TATCTGCAGTAATCATCCACGCTCGAGTTACCACC	
p.(L151F) F	CTGGTGGTAACTCGAGCGTTTATGATTACTGCAGATATTC	
p.(L151F) R	GAATATCTGCAGTAATCATAAACGCTCGAGTTACCACCAG	
p.(G198D) F	ATCCTCCCTTTCTTTCAGATACCCCAGGAATCATTG	
p.(G198D) R	CAATGATTCCTGGGGTATCTGAAAGAAAGGGAGGAT	
p.(G198A) F	TCCTCCCTTTCTTTCAGCTACCCCAGGAATCATT	
p.(G198A) R	AATGATTCCTGGGGTAGCTGAAAGAAAGGGAGGA	
