# Supplementary material for: Exonic CLDN16 mutations associated with familial hypomagnesemia with hypercalciuria and nephrocalcinosis can induce deleterious mRNA alterations
Source: BMC Med Genet. 2019 Jan 8;20:6. doi: 10.1186/s12881-018-0713-7 (PMC6325764; doi:10.1186/s12881-018-0713-7)
Supplement: Supplementary file 2 — Table S2. Bioinformatics predictions of splicing defects for CLDN16 exonic mutations and comparison with experimental results. (PDF 94 kb) [file 12881_2018_713_MOESM2_ESM.pdf]

Table S2. Bioinformatics predictions of splicing defects for *CLDN16* exonic mutations and comparison with experimental results.

Mutation	Exon	MutPred Splice		SPANR	Human Splicing Finder	Minigene result
		Result	Confident hypothesis	PSI	Confident hypothesis	
c.341G>A p.(R114Q)	2	SNV	---	Decreased	New ESS/ESE Site Broken	No effect
c.416C>T p.(A139V)	2	SNV	---	Increased	No significant alteration	No effect
c.421C>G p.(H141D)	2	SNV	---	No effect	ESE Site Broken	No effect
c.434T>C p.(L145P)	3	SNV	---	No effect	ESE Site Broken	No effect
c.446G>A p.(R149Q)	3	SNV	---	Decreased	ESE Site Broken	No effect
c.446G>T p.(R149L)	3	SNV	---	Decreased	ESE Site Broken	Truncated exon 3
c.452T>G p.(L151W)	3	SNV	---	Increased	No significant alteration	No effect
c.453G>T p.(L151F)	3	SNV	---	No effect	ESE Site Broken	Truncated exon 3
c.485G>T p.(G162V)	3	SAV	ESS Gain	No effect	ESE Site Broken	No effect
c.571G>A p.(G191R)	3	SNV	---	Decreased	ESE Site Broken/New 3′ SS	Exon 4 skipping
c.593G>A p.(G198D)	4	SAV	---	No effect	No significant alteration	Exon 4 skipping
c.593G>C p.(G198A)	4	SAV	Loss of 3' SS	No effect	No significant alteration	Exon 4 skipping

PSI, percentage of transcripts with exón incorporated; SNV, splicing neutral variant; SAV, splicing affecting variant; (---), no effect; ESS, exonic splicing enhancer; ESE, exonic splicing enhancer; SS, splice site
