# Supplementary material for: Exonic CLDN16 mutations associated with familial hypomagnesemia with hypercalciuria and nephrocalcinosis can induce deleterious mRNA alterations
Source: BMC Med Genet. 2019 Jan 8;20:6. doi: 10.1186/s12881-018-0713-7 (PMC6325764; doi:10.1186/s12881-018-0713-7)
Supplement: Supplementary file 3 — Figure S1. Precise nature of aberrant transcripts as deduced by DNA sequences and predicted exon fusions. (PDF 1807 kb) [file 12881_2018_713_MOESM3_ESM.pdf]

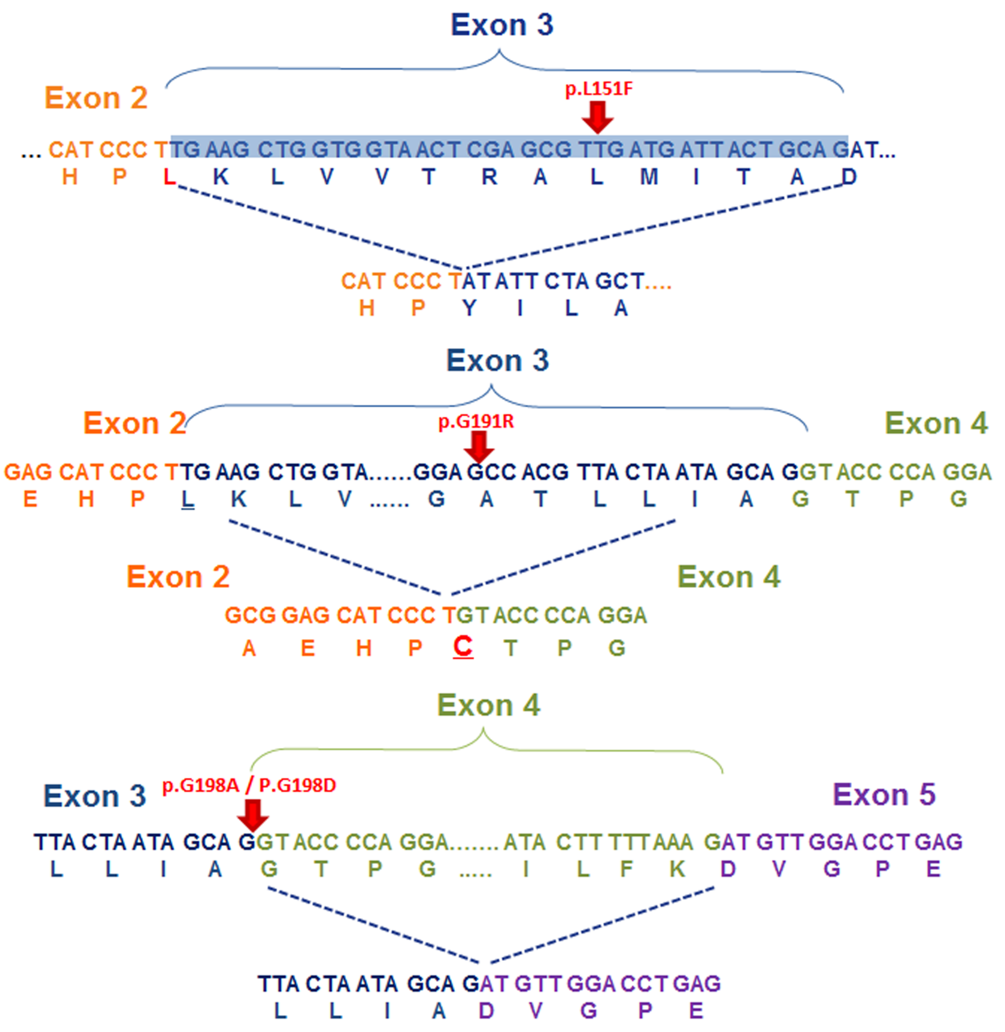

Fig. S3. Precise nature of aberrant transcripts as deduced by DNA sequences and predicted exon fusions. Arrows and brackets indicate the position of mutations and the nucleotides/amino acids lost with each mutation, respectively.
